# The association between playing professional American football and longevity

**DOI:** 10.1073/pnas.2308867120

**Published:** 2023-10-30

**Authors:** John Robert Warren, Gina Rumore

**Affiliations:** ^a^Institute for Social Research and Data Innovation, University of Minnesota, Minneapolis, MN 55455

**Keywords:** mortality, occupation, research design, football

## Abstract

Recent research concludes that professional American football players (hereafter, “football players”) live longer than American men in general, despite experiencing higher rates of chronic traumatic encephalopathy (CTE) and cardiovascular disease (CVD). This suggests that the longevity-enhancing benefits of playing football (e.g., physical fitness, money) outweigh the costs associated with CTE, CVD, and other longevity detriments of playing football. However, these surprising results may be the consequence of flawed research design. To investigate, we conducted two analyses. In analysis 1, we compared a) all professional American football players whose first season was 1986 or between 1988 and 1995 to b) a random sample of same-age American men observed as part of the National Health Interview Surveys in those same years selected on good health, at least 3 y of college, and not being poor. The exposure consists of playing one or more games of professional football; the outcome is risk of death within 25 y. In analysis 2, we use data on 1,365 men drafted to play in the (American) National Football League in the 1950s—906 of whom ultimately played professional football, and 459 of whom never played a game in any professional league. We estimate the association between playing football and survival through early 2023. In both analyses, we investigate differences between linemen and other position players. In contrast to most prior research, in both analyses, we find that linemen died earlier than otherwise similar men; men who played other positions died no earlier (or later).

## Significance

Professional American football—a major cultural, institutional, and economic force in American society in recent decades—has been publicly criticized for its negative impacts on the cardiovascular and brain health of players participating in the sport. Perhaps surprisingly, however, recent evidence suggests that the benefits of playing professional American football may outweigh the costs such that players live longer than other men. We conclude that this counterintuitive finding is due to flawed methodological designs of many prior studies. Using two stronger research strategies, we find no evidence of a positive association between playing football and longevity; indeed, linemen’s lives are shorter.

A robust body of research finds that professional American football players (hereafter, “football players”) live longer than men in the United States in general (1–7). On the surface, this finding might seem surprising given publicity and research about high rates of cardiovascular (8–12) and neuropsychological problems (many resulting from traumatic brain injuries) (13–18) among football players. On the other hand, football players are elite athletes, often earn very high salaries, and almost always have at least 3 y of college education—all factors that have strong positive influences on health and longevity. With only a handful of exceptions (19), the current body of evidence suggests that the longevity benefits of playing professional American football may outweigh the longevity costs of playing the game—resulting in longer lives for players.

Nearly all this evidence about the longevity consequences of playing football comes from research designs that contrast the mortality outcomes of football players with those of men who may not be comparable in important respects. In most cases, football players are compared to “American men in general” (4, 6, 7, 13, 20) or to elite athletes who played different sports (19, 21). In both cases, we are concerned about the validity of the comparison group.

Research that compares football players to men in the United States in general may be conflating “healthy worker effects” (6, 22–24)—a form of selection bias—with the actual benefits and harms of playing football. Men who—like football players—are healthy, relatively well educated, and able to hold jobs for extended periods of time are, on average, likely to live longer than men in general even when their actual jobs may have substantial negative effects on their health (25, 26). Football players may thus live longer than men in the United States in general because of selection factors (e.g., good health, education) that allow them to play football in the first place and to continue to play football (or to hold any job) at a professional level. At the same time, this research also implicitly ignores another important difference between football players and men in the United States in general: In recent decades, football players are disproportionately racialized as Black. Given racial disparities in early mortality (27, 28), previous research that compares football players to American men in general may understate the mortality advantages of playing football.

On the other hand, prior studies that compare football players to elite athletes who play other sports may suffer from a different form of selection bias: This research implicitly assumes that the social, economic, regional, racial, and cultural processes that influence who plays football (29, 30) are the same as the processes that influence who plays basketball, baseball, soccer, or other sports; this may or may not be true. As a result, it is not entirely clear whether differences in mortality outcomes between football players and other athletes are because of the sport itself or because of differences in the selectivity of who plays which sports. To properly assess the association between longevity and playing professional American football, we must compare football players to men who are as similar as possible with respect to racial, socioeconomic, health, and other factors that impact the timing of men’s death.

The balance between the harms and benefits of playing football for longevity may also depend on what position men play in the sport. For example, there is growing evidence that offensive and defensive linemen (hereafter, linemen) are especially likely to suffer health complications related to cardiovascular disease (CVD) (8–10, 31–33)—mainly due to rapid weight gain (beginning in college) and strength training (throughout their careers) in the absence of sufficient cardiovascular conditioning. There is also evidence that linemen are at elevated risk of experiencing concussions and chronic traumatic encephalopathy (CTE) compared to other players (34, 35). For this reason, we follow prior research (4, 31, 36, 37) in distinguishing linemen from other players in assessing the impact of playing football on longevity.

Is playing professional American football associated with men’s longevity? Does that association depend on playing position? We expect that we will—like a great deal of prior research—initially observe that football players live longer as compared to men in the United States in general. However, we hypothesize that this association will be greatly reduced or eliminated when football players are compared to American men who are similarly selective with respect to education, health, racial group membership, and other factors. Further, we hypothesize that our results will vary by playing position, such that linemen will fare worse than other position players with respect to longevity.

## Results

To test these hypotheses, we conducted two complementary sets of analyses. In analysis 1, we begin—like much prior research—by comparing the longevity of football players to the longevity of American men in general; we observe the latter group in the U.S. National Health Interview Survey (NHIS). We then repeat that comparison after restricting the NHIS sample to men who are comparable to football players with respect to education, health, race/ethnicity, and other factors. In analysis 2, we compare football players to men who were highly at risk of playing professional football but who never actually played. Here, we compare men who were drafted to play professional American football and who did play to men who were drafted to play football but who never played a single professional game. The “*Methods and Materials*” section describes how we constructed the requisite data for both analyses, as well as how we conducted both sets of analyses.

### Analysis 1.

Analysis 1 includes 21- to 25-y-old male NHIS respondents and first-year American professional football players observed in 1986 or between 1988 and 1995; 1987 is excluded because of a players’ strike. Table 1 reports descriptive statistics for key measures, separately for the sample of men in the NHIS (the first column) and for all football players (the third column). Comparing the two groups with respect to risk of death within 25 y of initial observation, Table 1 shows that 3.1% of football players died within 25 y as compared to 4.9% of same-age men in the United States in general. This replicates prior research; this is also where much prior research stops.

**Table 1.** Descriptive statistics, analysis 1

	Full NHIS sample	Restricted NHIS sample	All football players	Linemen	Other positions
Mortality					
Died within 25 y of initial observation (by age 46 to 50)	4.9%	2.3%	3.1%	5.3%	2.2%
Socioeconomic and health factors					
Completed 3 or more years of college	44.5%	100.0%	–	–	–
Excellent or very good health	77.4%	100.0%	–	–	–
Not in bed >2 d with illness, injury	80.4%	100.0%	–	–	–
Employed for pay by self or other	81.5%	100.0%	–	–	–
Family income is $25,000 or more	49.7%	100.0%	–	–	–
Number of observations	31,836	4,342	2,525	730	1,795

Note: Statistics pertain to 21 to 25-y-old male NHIS respondents and first-year American professional football players observed in 1986 or between 1988 and 1995. The full NHIS sample includes men aged 21 to 25 in the 1986 and 1988 to 1995 NHIS samples. The restricted NHIS sample includes only those men who completed 3 or more years old college, were in excellent or very good health, were not in bed with illness or injury for more than 2 d in the preceding year, were employed for pay, and had family incomes of $25,000 or more. “All Football Players” includes first-year American professional football players between the ages of 21 and 25 in each year between 1986 and 1988 to 1995. Linemen includes both offensive and defensive linemen; other positions include all other players. NHIS estimates are weighted by the sampling weight MORTWT.

However, professional American football players have almost always completed three or more years of college; are generally in very good health and lack serious illnesses or disabilities; are (by definition) employed; and (at least between 1986 and 1995) earned moderately high incomes. Only about 1 in 7 men in the United States were like football players in all these respects in these years. Thus, we restrict the NHIS sample to the subset of 4,342 same-age American men who completed at least 3 y of college; were in good health; were not confined to bed for more than 2 d in the preceding year with illness or injury; were employed; and earned family incomes of at least $25,000. When we compare this subset of NHIS men to football players, the differences in risk of early death are eliminated completely: As shown in Table 1, whereas 3.1% of football players died within 25 y, only 2.3% of comparable men in the United States did so.

However, as shown in the fourth and fifth columns of Table 1, football players’ risk of early death varies by position. Offensive and defensive linemen—perhaps because of an elevated risk of CVDs and CTE—have a relatively higher risk of early death than other football players. On the other hand, all other players have a relatively lower risk of early death.

**Fig. 1.**
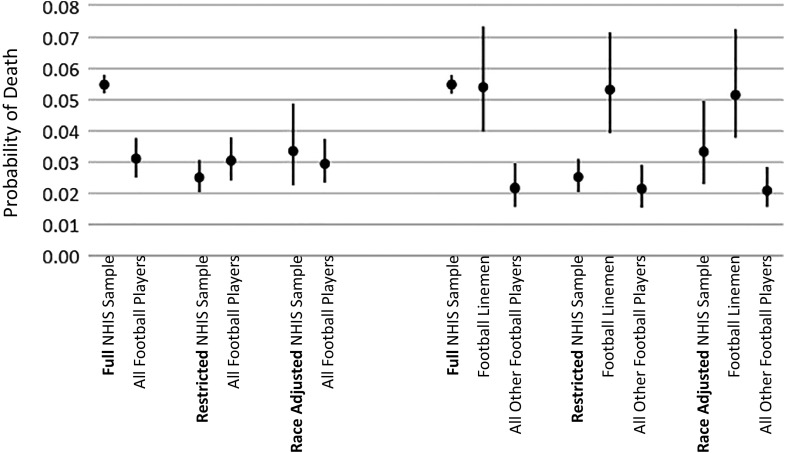
Estimated effect of playing American professional football on risk of death within 25 y, analysis 1. Note: Figures depict point estimates and 95% CIs derived from rare event logistic regression models of mortality status on indicators of having played football. The full NHIS sample includes men aged 21 to 25 in the 1986 and 1988 to 1995 NHIS samples. The restricted NHIS sample includes only those men who completed 3 or more years old college, were in excellent or very good health, were not in bed with illness or injury for more than 2 d in the preceding year, were employed for pay, and had a family income of $25,000 or more. The race-adjusted NHIS sample weights the restricted sample to reflect the racial/ethnic composition of professional football players. “All Football Players” includes first-year American professional football players between the ages of 21 and 25 in each year between 1986 and 1988 to 1995. Linemen includes both offensive and defensive linemen; other positions include all other players. NHIS estimates are weighted by the sampling weight MORTWT. All estimates are from models that adjust for year and age.

**Fig. 2.**
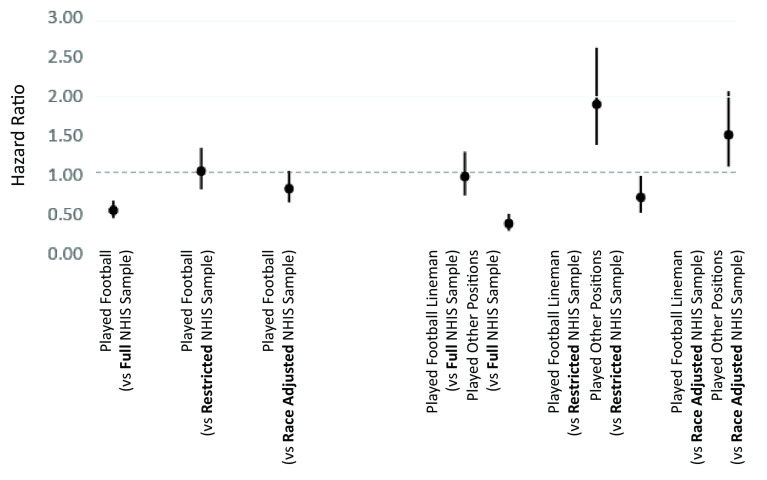
Estimated effect of playing American professional football on hazard of death, analysis 1. Note: Figures depict point estimates and 95% CIs derived from Cox proportional hazard models of mortality on indicators of having played football. The full NHIS sample includes men aged 21 to 25 in the 1986 and 1988 to 1995 NHIS samples. The restricted NHIS sample includes only those men who completed 3 or more years old college, were in excellent or very good health, were not in bed with illness or injury for more than 2 d in the preceding year, were employed for pay, and had a family income of $25,000 or more. The race-adjusted NHIS sample weights the restricted sample to reflect the racial/ethnic composition of professional football players. “All Football Players” includes first-year American professional football players between the ages of 21 and 25 in each year between 1986 and 1988 to 1995. Linemen includes both offensive and defensive linemen; other positions include all other players. NHIS estimates are weighted by the sampling weight MORTWT. All estimates are from models that adjust for year and age.

Figs. 1 and 2 depict results from logistic regression and event history models, respectively. Fig. 1 reports results of models predicting death within 25 y of first observation; the figure depicts predicted probabilities of death within 25 y, along with 95% CIs. Fig. 2 reports results of models of timing of death between first observation and 2019; the figure depicts hazard ratios and 95% CIs.

As shown in Figs. 1 and 2, football players were at substantially lower risk of early death as compared to same-age men in the United States in general. Adjusting for age and year, football players’ odds of surviving for 25 y beyond initial observation were 82% higher (OR = 1.82; 95% CI = 0.73 to 1.77; *P* < 0.001). In the event history models, football players’ hazard of death was markedly lower than that for same-age men in the United States in general (Hazard = 0.53; CI = 0.43 to 0.66; *P* < 0.001).

However, results are markedly different when we compare football players to the restricted subset of same-age men in the United States who were more like football players with respect to educational attainment, health, disability, employment status, family income, and racial composition. As shown in Figs. 1 and 2, the key associations are greatly reduced in magnitude and are no longer statistically significant. Net of age and year, football players’ odds of surviving for 25 y beyond initial observation were not significantly different than those of otherwise comparable men (OR = 1.14; 95% CI = 0.73 to 1.77; *P* = 0.559). In the event history models, football players’ hazard of death was not significantly different than for otherwise comparable men (Hazard = 0.81; CI = 0.63 to 1.04; *P* = 0.094).

As shown in the right-hand sides of Figs. 1 and 2, results differ markedly when we distinguish linemen from other position players. Comparing football players to same-age men in the United States in general, we see that nonlinemen are substantially less likely than men in the United States in general to experience early death. For them, and net of age and year, the odds of surviving for 25 y beyond initial observation were more than double those of men in the United States in general (OR = 2.59; 95% CI, 1.88 to 3.58; *P* < 0.001). In contrast, linemen appear no less (or more) likely to die within 25 y as compared to same-age men in the United States in general (OR = 1.03; 95% CI = 0.74 to 1.43; *P* = 0.869). Results from hazard models depicted in Fig. 2 are similar.

When we compare football players by position to the subset of men in the United States who more closely resemble football players—see the right-most set of results in Figs. 1 and 2—we see no significant differences between linemen or nonlinemen and otherwise similar men in their risk of death within 25 y (For linemen, OR = 0.63; CI, 0.38 to 1.05; *P* = 0.078; For other players, OR = 1.63; CI = 0.99 to 2.67; *P* = 0.05). However, results from the event history models (Fig. 2) show that—as compared to otherwise similar men—linemen have a higher hazard of death (Hazard = 1.49; CI = 1.09 to 2.05; *P* = 0.013), whereas other players have a lower hazard of death (Hazard = 0.55; CI, 0.74 to 0.89; *P* < 0.001). Note that nonlinemen’s advantage with respect to hazard of death is substantially smaller than in the model that compared them to men in general.

### Analysis 2.

As described in the “*Methods and Materials*” section, for analysis 2, we begin with all 1,463 men who were selected in the first 12 rounds of the National Football League (NFL) draft between 1950 and 1959. Our analytic file consists of the 1,365 of these men for whom we have measures of a) birthdate and b) number of professional games played. Of the 1,365 men, 906 played at least one professional game; 459 never played a single game in any professional league (in the United States or Canada); 497 were linemen (on offense or defense); and 868 played other positions.

Our key treatment measure is a binary indicator of whether men ever played one or more games of professional American football (regardless of which league they played in). We also consider both the intensity of the treatment (i.e., how many games men played) and heterogeneity in the effects of the treatment across playing positions. Our outcome measure is longevity, expressed as the number of days from a man’s being drafted into the NFL until his death.

Our analyses consist of descriptive analytic procedures and event history models of time to death. Because we are studying the entire population of men drafted in the first 12 rounds of the NFL drafts between 1950 and 1959, we do not employ inferential statistics (e.g., CIs, SEs, *P* values) in our analyses; assuming our model is correct, our point estimates represent true population parameters for men drafted in the first 12 rounds in this era.

In Table 2, we provide descriptive statistics for all measures, separately by playing position (linemen vs. all other positions) and by whether drafted men ever played in any professional football league. About 87% of men drafted to play in the NFL in the 1950s were deceased by February 1, 2023; this figure was somewhat higher for linemen and somewhat lower for other position players. Among deceased men, average age at death was lower for linemen as compared to other position players and lower for men who played one or more games as compared to men who never played. A key result in Table 2 is that among men drafted to be linemen, those who eventually played professionally died about 1.6 y earlier than those who never played.

**Table 2.** Descriptive statistics, analysis 2

Variable					Offensive and defensive linemen				Other position players			
	All drafted men				Played 0 games		Played 1+ games		Played 0 games		Played 1+ games	
	Avg./%	(SD)	Min.	Max.	Avg./%	(SD)	Avg./%	(SD)	Avg./%	(SD)	Avg./%	(SD)
Dead as of 2/1/2023	87.0%		0	1	89.4%		89.6%		85.8%		85.4%	
Age at death (if dead)	75.5	(13.1)	22.6	95.6	75.2	(13.1)	73.6	(13.1)	76.5	(13.5)	76.1	(12.8)
Professional games played	39.9	(51.8)	0	255	–		59.4	(52.8)	–		60.4	(53.5)
Age at draft	22.4	(1.5)	15.7	38.6	22.5	(1.6)	22.4	(1.4)	22.7	(1.9)	22.3	(1.2)
Year of draft	1954.4	(2.9)	1950	1959	1954.2	(2.8)	1954.4	(2.9)	1954.1	(2.8)	1954.6	(2.9)
Round of draft	6.3	(3.5)	1	12	8.4	(3.0)	5.8	(3.1)	8.4	(2.8)	5.0	(3.3)
N	1,365				199		298		260		608	

Note: Analysis consists of all 1,365 men selected in the first 12 rounds of the NFL draft between 1950 and 1959 for whom we have measures of a) birthdate and b) number of professional games played.

**Fig. 3.**
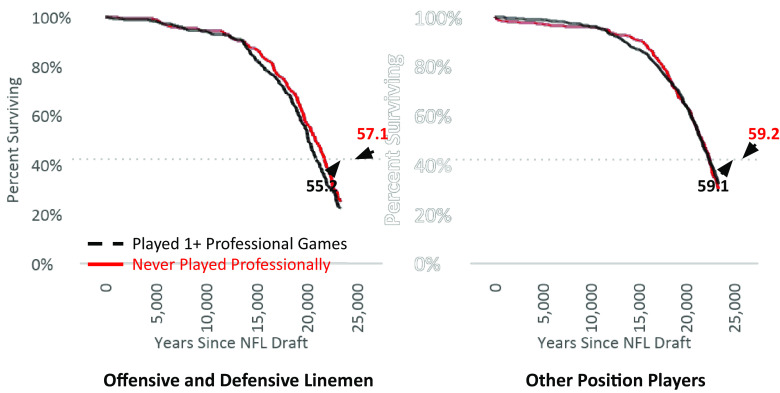
Rates of survival since NFL draft, by playing position and games played, analysis 2. Note: Analysis consists of all 1,365 men selected in the first 12 rounds of the NFL draft between 1950 and 1959 for whom we have measures of a) birthdate and b) number of professional games played. The figures above also report the median number of years lived after being drafted into the NFL, separately by playing position and number of games played (0 vs. more than 0). We do not report CIs, SEs, or other information about statistical uncertainty because the data we use contain the entire population of drafted men—not a sample from that population.

Fig. 3 depicts age-specific survival rates since the date on which men were drafted into the NFL, separately by playing position (linemen vs. other players) and number of professional games played in any league (zero vs. one or more). Whereas 100% of men were alive on their draft day, only about one-fourth of them were alive 64 y later. There are no meaningful differences between the survival curves of drafted nonlinemen who played vs. drafted nonlinemen who never played. In contrast, Fig. 3 shows that men drafted to be linemen died sooner if they played one or more professional games. For example, as reported in the figure, the median number of years from NFL draft to death was 57.1 for linemen who never played a single game but nearly 2 y lower (55.2) for linemen who played one or more games.

To account for the possibility that our descriptive results above are misleading because of systematic differences between groups of drafted men with respect to how old they were when they were drafted, the year in which they were drafted, and/or the round in which they were drafted, we estimate a series of multivariate Cox proportional hazard models. Drafted men who played one or more games may have been older on draft day (thus reducing their rate of survival after draft day) or they may have tended to be drafted earlier in the 1950s (such that secular increases in life expectancy may account for our descriptive results). Our models express the impact of playing American professional football on timing of death net of age at draft, year of draft, and draft round.

As shown in Table 3, we continue to see that among men drafted to play nonlinemen positions, playing professional football had little relationship with their longevity. This is true when we distinguish players from nonplayers (panel A on Table 3) and when we differentiate players according to how many games they played in their careers (panel B). In contrast, results from the multivariate event history models continue to indicate that among men drafted to be linemen, those who eventually played one or more games were at increased risk of death. Panel B of Table 3 indicates that the greatest risk was among linemen who played a moderate number of games (i.e., who were in the middle two quartiles of number of games played). As we elaborate in the *Discussion* below, we suspect that this is a variant of the “healthy worker effect.”

**Table 3.** Hazard ratios, analysis 2

	All drafted men	Offensive and defensive linemen	Other position players
Panel A. played (vs. did not play)			
Played 1+ games	1.04	1.14	1.00
Age at draft	1.07	1.04	1.09
Year of draft	1.01	1.00	1.01
Round of draft	0.99	1.00	0.98
Panel B. Quartile of games played (vs. did not play)			
Played, lowest quartile # of games	1.00	1.04	0.97
Played, 2nd quartile	1.02	1.14	0.98
Played, 3rd quartile	1.11	1.32	1.02
Played, highest quartile # of games	1.03	1.08	1.06
Age at draft	1.07	1.04	1.09
Year of draft	1.01	1.00	1.01
Round of draft	0.99	1.00	0.98

Note: Analysis consists of all 1,365 men selected in the first 12 rounds of the NFL draft between 1950 and 1959 for whom we have measures of a) birthdate and b) number of professional games played. We do not report CIs, SEs, or other information about statistical uncertainty because the data we use contain the entire population of drafted men—not a sample from that population.

## Discussion

Previous research has typically concluded that playing professional American football is associated with longer life expectancy; this is perhaps a surprising finding given relatively higher rates of cardiovascular problems and CTE among former players. We wonder whether this counterintuitive conclusion may be due to weaknesses in the designs of prior research. Professional football players are not a random or representative subset of all American men, and so it is inappropriate to compare them to American men in general. Professional football players are highly athletic, they have nearly always attended at least some college, and they are (at least initially) healthy enough for paid work. Even absent any longevity effects of playing, these systematic differences between professional football players and other men should lead to higher life expectancy among players than among American men in general.

Like prior research, we ask whether playing professional American football is associated with longevity. Unlike most previous investigations, we find that playing American professional football is associated with shorter the life expectancy among linemen and has little relationship with life expectancy among other players. In analysis 2, for example, we find that men drafted to be linemen died about 2 y earlier if they played one or more games as compared to drafted linemen who never played. Is this difference in life expectancy small or large? For context, this 2-y difference in longevity is about two-thirds the size of the effect of getting a bachelor’s degree (38) and about two-fifths the size of the effect of being female (39).

One basic methodological point for this field of research is that it is imperative to employ proper comparison groups to account for the selectivity of men who are at risk of playing professional American football. “American men in general” is not a proper comparison group; nor (we suspect) are men who played other sports professionally. Men at risk of playing professional football are selective with respect to education, health, race/ethnicity, and other factors that are consequential for mortality.

Although our designs are, we argue, methodologically stronger than in prior studies, our research is still limited in several ways. First, like prior research, our results only speak to the effects of playing professional football; collectively, we know very little—but not quite nothing (6)—about the longevity consequences of playing recreational, high school, or college football. Second, our results say nothing about the quality of life experienced by men who formerly played professional football. Although we find no longevity effects of playing football among nonlinemen, for example, there may still be sizable effects on later-life functional limitations, pain, cognitive impairments, and other outcomes among these men. Third, our research—like all prior research—uses observational designs. Although we argue that our estimates—especially from analysis 2—come closer to causal estimates, caution is warranted in making causal claims. Fourth, our results say nothing about heterogeneity in effects by race/ethnicity. Racism in postcareer health care, financial institutions, or other settings may mean that white players are better able to avoid longevity penalties associated with having played professional football. We hope that future research will improve on our knowledge in all these ways.

## Materials and Methods

### Analysis 1.

Analysis 1 compares the mortality outcomes of two groups of men in the United States. The first group includes 21- to 25-y-old men who were first-year (“rookie”) players in the NFL in 1986 or in any year between 1988 and 1995; we exclude 1987 because a players’ strike caused the league to use replacement players who may not be typical of all players. The second group includes 21- to 25-y-old men who were respondents to the cross-sectional NHIS (40) in 1986 or in any year between 1988 and 1995; the NHIS includes a randomly selected, nationally representative sample of community-dwelling individuals in the United States in each year. To replicate prior research, we begin by comparing the risk of early death of a) football players to b) same-age men in the United States in general. We then compare the risk of early death of a) football players to b) same-age men in the United States who are like football players with respect to educational attainment, current employment status, income, health, disability status, and racial/ethnic composition. We argue that the latter comparison better represents the association between playing football and risk of early death. In other words, we assume that football players were employed, college educated, healthy, not disabled, and earning at least moderate salaries at the outset of their playing careers, and we construct a control sample that has those characteristics at the same young ages; we also equate the two groups with respect to racial/ethnic composition. Finally, we make these same comparisons after distinguishing linemen from other players.

Information about football players is derived from the website Pro Football Archives (41). The site includes comprehensive information about every person who ever played in the NFL, including date of birth, date of death, years played in the NFL, number of games played in the NFL, and position played. Our analyses include all 2,525 players who played at least one game in the NFL, whose initial season was 1986 or between 1988 and 1995, and who were between the ages of 21 and 25 in that season. From information about initial year played in the NFL and year of death, we construct measures indicating whether players survived for 25 y beyond their initial NFL season. Event history analyses model risk of death between the year of players’ initial NFL seasons and 2019.

Information about men in the United States in general is derived from 1986 and 1988 through 1995 NHIS interviews. Our analyses include the 31,836 men who were between ages 21 and 25 in any of those NHIS years. Records include information about men’s age; education attainment (0 = completed 2 y of college or less; 1 = completed 3 y of college or more); current employment status (0 = not employed; 1 = employed); family income (0 = less than $25,000; 1 = $25,000 or more); self-assessed overall health (0 = good, fair, or poor health; 1 = excellent or very good health); and disability status (0 = spent 2 or fewer days in bed in the preceding year due to illness or injury; 1 = spent 3 or more days in bed). Records for NHIS respondents to the 1986 and 1988 through 1995 surveys have been linked by the National Center for Health Statistics to the National Death Index (42), which includes date of death; deaths are observed through 2019. From information about survey year and date of death, we construct a measure indicating whether men survived for 25 y beyond their NHIS survey. Event history analyses model risk of death between the year of men’s NHIS survey and 2019. To adjust for racial/ethnic composition, we reweight the NHIS data to cause it to reflect the approximate racial/ethnic distribution of football players in this era (43). All analyses use the NHIS sampling weight MORTWT, which accounts for survey nonresponse and selective eligibility for linkages to the National Death Index.

**Fig. 4.**
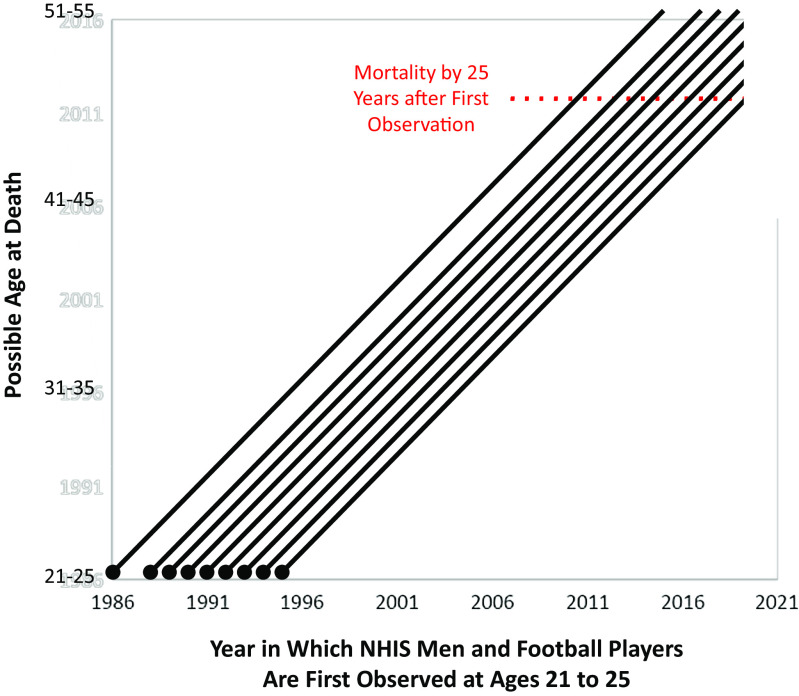
Years of observation and possible ages at death, analysis 1. Note: None.

For clarity, Fig. 4 depicts the possible ages at death of men who were first observed in 1986 or between 1988 and 1995. As shown in the figure, for all men we observe risk of death for at least 25 y after initial observation.

Our analyses of risk of death within 25 y from first observation—that is, from initial NFL season or NHIS survey—use rare event logistic regression models (44). Analyses of time to death between initial observation and 2019 use Cox proportional hazard (event history) models. All models include an indicator of whether men played professional American football; they also adjust for age (which ranges from 21 to 25) and calendar year of first observation. Our focus is on the magnitude, direction, and statistical significance of the coefficient for the indicator of whether men played football. We use 2-sided hypothesis tests and an a priori level of significance of α = 0.05. All analyses are executed using Stata (version 14.2, StataCorp).

### Analysis 2.

Our analytic sample for analysis 2 begins with all 1,463 men who were drafted to play in the NFL in the first 12 rounds of the 1950 and 1959 drafts; in most of these 10 y, there were 12 NFL teams, although in some years, there were 13 teams, and, in some years, there was a bonus first-round pick for a randomly selected team. We only considered the first 12 draft rounds for reasons of cost; as described below, determining birth and death dates for drafted men who never played took a great deal of time and effort.

We obtained information about each drafted man’s name, playing position, college football career, draft year/position/team, and number of NFL games eventually played from the sports information website Stathead.com. For men who ultimately played at least one game in the NFL, we merged records from the sports information website ProFootballArchives.com to obtain exact date of birth and (when applicable) exact date of death; note that because we initially linked records to ProFootballArchives.com in early 2022, no player deaths were recorded in ProFootballArchives.com after January 2022.

To obtain information about exact dates of birth and death for men who were drafted but who never played a single game in any professional league and information about deaths occurring after January of 2022 among men who did play one or more professional games, we used a team of trained undergraduate research assistants to conduct archival research.

Our team of undergraduate research assistants was trained to use various online resources to obtain a) exact dates of birth and death for men who were drafted between 1950 and 1959 to play in the NFL but who never played in a single professional game and b) information about deaths occurring after January of 2022 among men who did play one or more professional games. These online resources included (among others) obituaries, newspaper articles, genealogical websites like FindaGrave.com and Ancestry.com, Social Security claims files, and state birth and death records.

Our research assistants began with known information about men—including their name, approximate birth year (i.e., a man drafted in 1950 who played 4 y of football in college would typically have been born in about 1928); position played; and college attended. They did not conclude that they had obtained exact birth or death dates from online archival sources unless they could confidently conclude that the information pertained to exactly the man who was drafted. For example, an online obituary would need to mention that the deceased man was drafted to play in the NFL in the year listed on Stathead.com for the research assistant to use the birth and death dates included in that obituary. In some cases, this required careful triangulation: For example, a college football team’s website might identify the man’s exact birthdate and the year he was drafted; a genealogical source for a man with the same name and exact birthdate might then provide an exact death date; and an obituary for a man with those same birth and death dates might mention the college he attended. Only when research assistants were highly confident that they had identified information for the same man they were researching were they permitted to stop searching and record birth and/or death dates.

About 8% of men who were drafted to play in the NFL in this era never ultimately played in even one NFL game but did go on to play in the Canadian Football League (CFL); a very small number played in other professional football leagues. Men who played in Canadian or other professional leagues were treated as having played professional American football. For drafted men who never played in the NFL, research assistants thus also used online resources to determine whether the men ever played one or more games in the CFL or other professional leagues and (if they did) how many games they played.

All research assistants went through training in the responsible conduct of research (even though all the data are from public records), and all were required to complete training sessions, conducted by the authors, about how to rigorously and reliably search for drafted men in online archives. All research assistants then searched for information about the same set of 25 men as a training exercise; the authors had already obtained information for those 25 men. Only research assistants who demonstrated very high levels of accuracy in researching those 25 men were permitted to continue with the project.

Of the 1,463 drafted men, 794 were determined to have played one or more professional American football games in the NFL; 133 were determined to have played one or more games in another professional football league (most in the CFL); and 536 were determined to have never played even one professional football game. Of the 927 men who played American professional football in any league, we obtained birth dates for all but seven; of the 920 professional players with birth dates, we obtained death dates for 800 (87%) and the other 120 (17%) are presumed to have still been alive as of late February 1, 2023. Of the 536 men who never played a single game in any professional league, we obtained birth dates for all but 77; of the 460 such men with birth dates, we obtained death dates for 401 (87%) and the other 59 (17%) are presumed to have still been alive as of late February 1, 2023. This means that we lack birth dates for 84 men: 0 NFL players, seven men who played in other professional leagues, and 77 drafted men who never played professional football; these men are excluded from our analyses because we cannot confirm that we obtained valid outcome measures for them. We also exclude 14 cases in which we are not able to determine how many games players played. Note that in the small number of cases in which we obtained only birth year (but not month or day), we assumed that men were born on July 1 of that year; this assumption had no meaningful impact on our substantive findings. In the end, we are left with 1,365 cases in our analysis file.

Our key treatment measure is a binary indicator of whether men ever played one or more games of American professional football. As noted above, we also consider both the intensity of the treatment (i.e., how many games men played) and heterogeneity in the treatment effect across playing positions. Our outcome measure is longevity, expressed as number of days from players’ NFL draft until their death; the small number of men who had not died by February 1, 2023, are thus censored on the outcome measure but are still included in our event history models. Besides the treatment, the only other covariates included in our models are age (in days) on draft day, year of draft, and draft round.

In analysis 2, we rely primarily on descriptive statistics. However, to estimate the impact of playing professional American football on longevity, we estimate a series of Cox proportional (event history) models using the streg suite of commands in Stata 14.2.

Because we are studying the entire population of men drafted in the first 12 rounds to play in the NFL between 1950 and 1959, we do not employ inferential statistics (e.g., CIs, SEs, *P* values) in our analyses; our point estimates represent true population parameters for men drafted in this era.

## Data Availability

Data from the National Health Interview Survey (NHIS) are publicly available (45). Data (birth and death dates, playing dates, and positions) for football players have been deposited in https://www.rob-warren.com/football.html (46).
